# Habitual levels of higher, but not medium or low, impact physical activity are positively related to lower limb bone strength in older women: findings from a population-based study using accelerometers to classify impact magnitude

**DOI:** 10.1007/s00198-016-3863-5

**Published:** 2016-12-13

**Authors:** K. Hannam, K. C. Deere, A. Hartley, U. A. Al-Sari, E. M. Clark, W. D. Fraser, J. H. Tobias

**Affiliations:** 1 Musculoskeletal Research Unit, University of Bristol School of Clinical Sciences, Southmead Hospital, Bristol, BS10 5NB UK; 2 University of East Anglia, Norwich, UK

**Keywords:** G-force, Hip BMD, Tibial pQCT

## Abstract

**Summary:**

This study assessed the effect of accelerometry-measured higher impacts resulting from habitual weight-bearing activity on lower limb bone strength in older women. Despite higher impacts being experienced rarely in this population-based cohort, positive associations were observed between higher vertical impacts and lower limb bone size and strength.

**Introduction:**

We investigated whether the benefit of habitual weight-bearing physical activity (PA) for lower limb bone strength in older women is explained by exposure to higher impacts, as previously suggested by observations in younger individuals.

**Methods:**

Four hundred and eight women from the Cohort for Skeletal Health in Bristol and Avon (COSHIBA), mean 76.8 years, wore tri-axial accelerometers at the waist for a mean of 5.4 days. *Y*-axis peaks were categorised, using previously identified cutoffs, as low (0.5–1.0 g), medium (1.0–1.5 g), and higher (≥1.5 g) impacts. Mid and distal peripheral quantitative computed tomography scans of the tibia and radius were performed, as were hip and lumbar spine Dual X-ray Absorptiometry (DXA) scans. Regressions between (log transformed) number of low, medium and high impacts, and bone outcomes were adjusted for artefact error grade, age, height, fat and lean mass and impacts in other bands.

**Results:**

Eight thousand eight hundred and nine (4047, 16,882) low impacts were observed during the measurement week, 345 (99, 764) medium impacts and 42 (17, 106) higher impacts (median with 25th and 75th quartiles). Higher vertical impacts were positively associated with lower limb bone strength as reflected by cross-sectional moment of inertia (CSMI) of the tibia [0.042 (0.012, 0.072) *p* = 0.01] and hip [0.067 (0.001, 0.133) *p* = 0.045] (beta coefficients show standard deviations change per doubling in impacts, with 95 % confidence interval). Higher impacts were positively associated with tibial periosteal circumference (PC) [0.015 (0.003, 0.027) *p* = 0.02], but unrelated to hip BMD. Equivalent positive associations were not seen for low or medium impacts.

**Conclusions:**

Despite their rarity, habitual levels of higher impacts were positively associated with lower limb bone size and strength, whereas equivalent relationships were not seen for low or medium impacts.

**Electronic supplementary material:**

The online version of this article (doi:10.1007/s00198-016-3863-5) contains supplementary material, which is available to authorized users.

## Introduction

Physical activity (PA) is thought to have a positive effect on bone health throughout the life course, through maximising peak bone mass in childhood and adolescence and minimising age-related bone loss in older adults. Research efforts are now increasingly focussed on optimising the non-pharmacological benefits of activity by determining optimal type, magnitude and duration of activity to maximise and maintain bone strength to ultimately reduce fracture risk. Interventional studies suggest that weight-bearing activities producing high vertical impacts improve lower limb bone strength in children and adolescents [1], pre-[2] and post-menopausal women [3] and older men [4]. The suggestion that the skeleton is primarily influenced by high impacts is consistent with laboratory studies indicating a dose response between strain level and bone formation [5]. Suppression of bone turnover may also contribute to the positive effects of mechanical bone loading, as exemplified by findings that C-terminal telopeptide (β-CTX) was reduced following an exercise intervention in older obese adults [6], and β-CTX levels in athletes decrease following exercise [7], although other studies show conflicting findings [8].

Observational studies also suggest that the amount of day-to-day participation in high-impact PA is positively related to lower limb bone strength, based on PA questionnaires in which different activities are graded according to impact level [9, 10]. Accelerometers provide more objective measures of PA exposure. However, in generating counts per minutes, widely used actigraph accelerometers use proprietary software which combines movement frequency and intensity, rather than evaluating peak acceleration magnitude per se. To define PA in terms of impacts to which the skeleton responds, the Newtest device was developed to measure the number of acceleration peaks according to *g* level. This starts sampling vertical accelerations each time these exceed a threshold of 0.3 g, following which the maximum acceleration value is identified and recorded, representing the acceleration peak for a given movement [11]. Using this device, the benefits of weight-bearing PA for hip BMD in premenopausal women were found to be explained by impacts beyond 3.9 g [12]. Similarly, in adolescents from the Avon Longitudinal Study of Parents and Children (ALSPAC), relationships between PA and hip BMD and tibial geometry were solely explained by vertical impacts beyond 4.2 g, despite their rarity (e.g. median of 7.8 per day in females) [13, 14]. However, to what extent equivalent relationships are observed in older populations are currently unknown.

Improvements in technology have since made it possible to store 7 days of continuous accelerometer recordings, enabling acceleration peaks to be identified retrospectively by applying data processing algorithms to raw data [15]. In addition, if acceleration peaks are recorded in time sequence, artefacts and errors can be identified more readily [15]. Applying this method to a population-based sample of older individuals, we recently found that virtually no acceleration peaks occur beyond 2 g, reflecting their lack of participation in high-impact PA. Therefore, although a 1.5 g threshold can be applied in this group to define relatively high vertical impacts, application of the conventional 4-g osteogenic threshold does not appear to be feasible. That said, osteogenic thresholds as defined in younger populations may not be applicable to older individuals, in whom lower levels of impacts may produce equivalent strains to those resulting from higher impacts in younger people, due to a weaker skeleton.

In the present study, we investigated whether skeletal benefits of habitual weight-bearing PA can be explained in terms of exposure to vertical impacts >1.5 g in a population-based cohort of older women, despite the rarity of these impacts and the relatively low threshold used to define higher impacts. Specifically, we examined (i) whether habitual exposure to higher, but not medium or low, vertical impacts as defined in this way are positively related to lower limb bone strength, (ii) whether relationships between higher impacts and lower limb bone strength are explained by changes in geometry and/or BMD, and (iii) whether alterations in bone formation or resorption underlie these associations as reflected by measurement of N-terminal propeptide of type I collagen (P1NP) and β-CTX, respectively.

## Materials and methods

### Study design

**Fig. 1:**
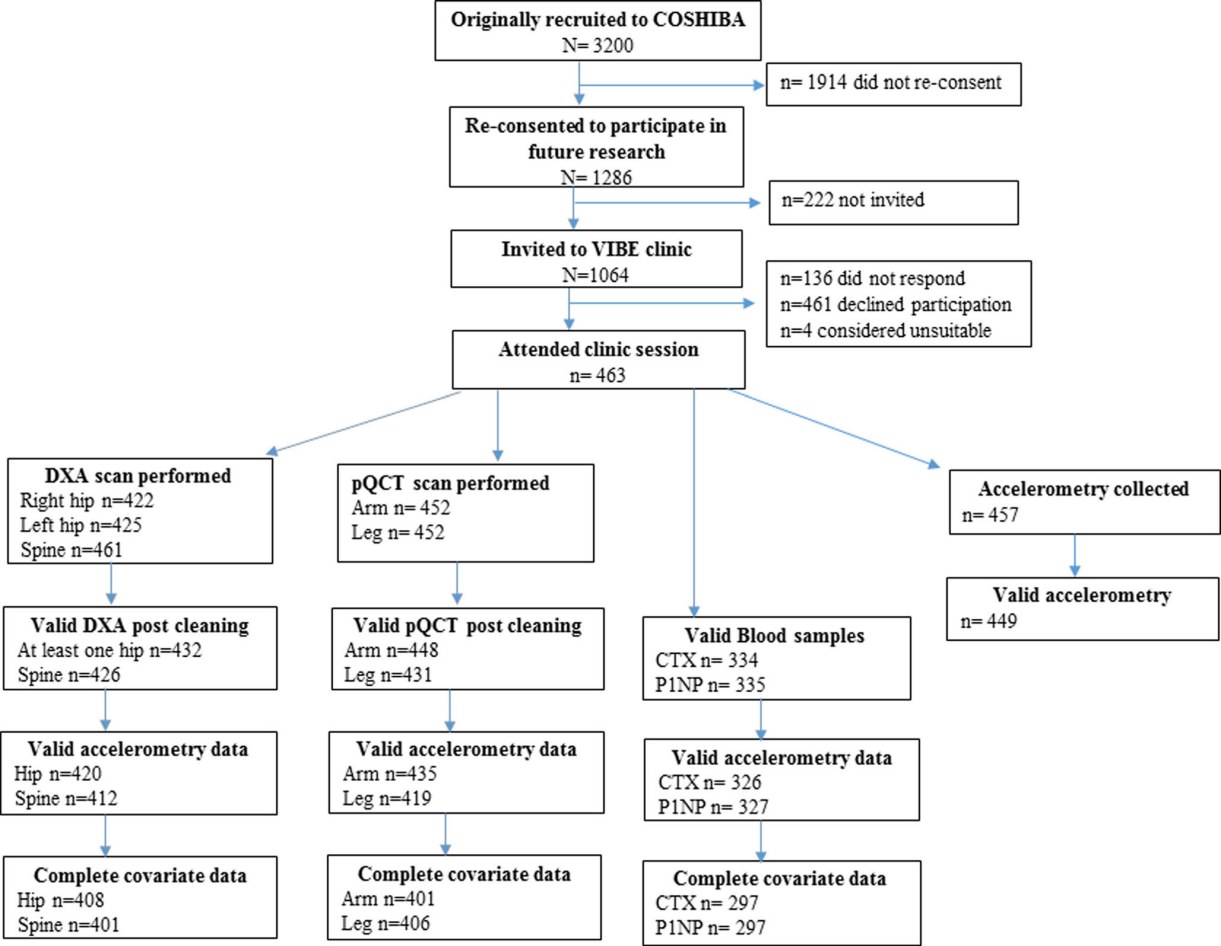
Flow diagram showing the recruitment of participants to the study

The VIBE study included participants from the Cohort for Skeletal Health in Bristol and Avon (COSHIBA) on which the present analysis is based. COSHIBA is a population-based cohort of 3200 women who were originally recruited in the Bristol and Avon area during 2007–2009 from GP surgeries with the sole-entry criteria of date of birth falling between 1 January 1927 and 31 December 1942 [16, 17]. Of the original 3200 COSHIBA participants who remained resident in the Bristol and Avon area, 1286 consented to be contacted about future research studies in 2014 and were eligible for inclusion in the VIBE study. Eligible participants were subsequently invited in alphabetical order, stopping after 1064 by which time available clinic assessment slots had been filled (see Fig. 1). COSHIBA participants attended VIBE clinic sessions throughout 2015 during which measures of skeletal health were obtained, immediately following which the 7-day accelerometry data collection was commenced. An explanation of the assessments followed by written informed consent and a verbal consent check before each procedure was obtained from every participant. The study was approved by the National Research Ethics Committee (14/SW/0138) in Bristol (South West-Frenchay).

### Accelerometry

Participants who consented to 7-day PA monitoring were provided with a GCDC X15-1c triaxial accelerometer (Gulf Coast Data Concepts, Waveland, Mississippi) along with a size-specific elasticated belt, a daily time log (recording time accelerometer was taken on/off and whether the day was normal in terms of activity) and a stamped addressed package along with written and verbal instructions. Participants were instructed to wear the accelerometer securely positioned over their right hip pointing toward the centre of their body for 7 consecutive days, only to be removed for sleeping, washing and swimming. Accelerometers were configured by the research team prior to participant use with a sampling frequency of 50 Hz. Raw accelerometry data were imported to Stata 13 (StataCorp, College Station, TX) for standardised processing using custom designed code (explained in detail elsewhere [15]). Briefly, accelerometry data were cleaned to remove movement artefacts and non-wear time. A given day was excluded if less than 10 h of valid recording time was obtained, to ensure that each day’s recording was representative of the total amount of PA undertaken [15]. Sensitivity analyses were performed with the restriction of only those with ≥3 valid days, as previously applied [15]. As previously described, *Y*-axis peaks were identified based on accelerations that were higher than the preceding and subsequent readings and grouped into three bands to reflect low (≥0.5 to <1.0 g), medium (≥1.0 to <1.5 g) and high (≥1.5 g) impact (movement ≤0.5 g were deemed sedentary activity and excluded) over and above 1 g of earth’s gravitational force [15]. Activity data were normalised based on 7 valid days (≥10-h recording time) of 14 h.

### pQCT

All COSHIBA participants who attended the outcome assessment clinic were offered a peripheral quantitative computed tomography (pQCT) at the tibia and radius using the Stratec XCT2000L (StraTec Medizintechnik, Pforzheim, Germany), which was performed as per standard operating procedures. The right arm (4 and 60 % of the radius) and leg (4, 50 and 66 % of the tibia) were scanned unless there was a history of recent fracture, or excessive tremor or metal pins and plates were present. Measurements were obtained for cortical BMD (BMD_c,_ mg/cm^3^) and trabecular BMD (BMD_t,_ mg/cm^3^) using XCT custom software. Additional pQCT variables were derived from a circular ring model including cortical thickness (CT, mm); periosteal circumference (PC, mm); cross-sectional moment of inertia (CSMI, mm^4^) and strength strain index (SSI, mm^3^), using a threshold of 350 mg/cm^3^ to define bone. Trabecular BMD measurements were taken from the 4 % site of the tibia and radius and all other measurements were taken or derived from results at the 60 % site for the radius and 50 % site for the tibia. All scan images were visually checked at the time the scan was performed and assigned a grade (0–5) based on the extent of artefact present. A score of 0 indicated no artefact, minimal artefact (score of 1), small (2), moderate (3), large—no analysis possible but features are still recognisable (4), and large—recognition of anatomical features not possible (5). The clinic workers were advised to repeat the scan if appropriate where a grade 3–5 was assigned. Analyses were based on scans with artefact scores of 0–3, of which 96.5 and 80.2 % were graded 0–2 for tibial and radial scans, respectively. Repeat tibial pQCT scans were performed in 20 COSHIBA participants within 1 month of the original scan. Within subject coefficients of variation at the 50 % site were 2.46 % for PC, 0.93 % for BMD_c_, 2.97 % for CT, 8.27 % for CSMI, 5.49 % for SSI and 5.87 % for BMD_t_.

### Anthropometric and DXA variables

Dual x-ray absorptiometry scan (DXA) was performed on a GE Healthcare Lunar Prodigy. Standing height was measured to the nearest millimetre using a Harpenden stadiometer (Holtain Ltd., Crymych, UK) and weight was measured to the nearest 50 g using Tanita weighing scales (Tanita UK Ltd., Uxbridge, UK). Consenting participants who were able to transfer onto the DXA scan bed unaided underwent a total body (generating fat and lean mass (FM and LM, kg), lumbar spine (L1–L4 (LS) BMD, g/cm^2^) and left and right hip scans (generating total hip (TH) and femoral neck (FN) BMD, g/cm^2^). The manufacturer’s automated advanced hip analysis (AHA) software was used to derive minimum neck width (MNW, mm) and cross-sectional moment of inertia (CSMI, mm^4^). For the purpose of analyses, the right hip results were used unless of joint replacement, recent fracture or significant artefact. DXA scans were cleaned using a systematic approach to correct for positioning errors, where possible, and identification and coding of minor and major artefacts. A substantial proportion of lumbar scans were found to have significant lumbar spondylosis, necessitating the exclusion of one or more lumbar vertebrae, which was performed in preference to excluding the whole lumbar spine scan. Repeat total body and hip DXA scans were performed in 20 COSHIBA participants within 1 month of the original scan. Within subject coefficients of variation were 1.65 % for FM, 1.48 % for LM, 1.51 % for TH BMD, 1.86 % for FN, 1.49 % for MNW and 5.96% for CSMI.

### Bone turnover markers

Participants were eligible to provide a blood sample at the clinic session provided they gave informed consent, had not been diagnosed with a bleeding disorder, were not currently taking anti-coagulant drugs and had not been recently diagnosed with anaemia, hepatitis B and C or HIV/AIDS positive. Participants were requested to fast for at least 4 h prior to their appointment and last time since eating/drinking was recorded. All samples were centrifuged at the study centre laboratory as soon as possible after obtaining the sample (≤20 min) and labelled aliquots were stored at −80 °C. Samples were then sent for analyses of β-CTX and propeptide of type I collagen (P1NP) at the Bioanalytical facility at the University of East Anglia. Electrochemiluminescence immunoassays (ECLIA) on a Cobas 1600i analyser (Roche Diagnostics, Lewes, UK) were used, with detection limits of 0.01 μg/L (β-CTX) and 8 μg/L. (P1NP); inter and intra-assay coefficients of variation (CVs) were <4.0 % across the working range of both assays.

### Additional confounders

On attending the research clinic, participants were also asked to complete a questionnaire asking about potential confounders which were also analysed as part of the study. These included self-reported health status (5-point scale from very poor to very good), co-morbidities potentially influencing PA participation (i.e. respiratory disease, cardiovascular disease, arterial disease, stroke, Parkinson’s disease and arthritis), which were then summed, and socioeconomic status based on their main occupation (and that of their spouse if married) during their working life. Each occupation was assigned a SOC90 (Standard Occupation Classification) code and collapsed to nine groups, the highest SOC90 code between the participant and spouse being assigned where both occupations were provided. Whether subjects were currently taking bone active agents (i.e. bisphosphonates, denosumab or strontium) was also recorded, as was the extent of PA participation below 18 years of age, and between age 18–29.

### Statistical analyses

Descriptive data on participant characteristics, accelerometry, DXA, pQCT and bone turnover markers were expressed as means and medians with standard deviations (SD) and the 25th and 75th percentiles. Accelerometry data were positively skewed and thus log transformed for regression analyses, which led to normalisation of data as assessed by visual inspection of histograms. Multivariate linear regression analyses were performed to explore associations between the number of *Y*-axis peaks, and DXA, pQCT and bone turnover markers, in the study population as a whole. A three-band model was used whereby 0.5 < *g* ≤ 1.0, 1.0 < *g* ≤ 1.5 and *g* > 1.5 counts were entered simultaneously, after checking model parameters (*r* ^2^ and Akaike information criterion (AIC)). All outcome variables were standardised and results presented indicate a SD change in the outcome variable per doubling in activity. Three models were used to explore associations between activity and bone outcome measures, a minimally adjusted model including only age as a potential confounder, a second model including age, height, fat and lean mass, and a third model incorporating additional adjustment for impacts in other bands. Analyses of radial and tibial pQCT data also included adjustment for error grading at the relevant site in both models, and those for bone turnover markers whether samples were am/pm. Further models were included in the analyses of tibial pQCT adjusting for β-CTX, P1NP, socioeconomic status and health status as assessed by questionnaire.

## Results

### Subject characteristics

1064 COSHIBA participants were invited to the VIBE research clinic of whom 463 attended a clinic session and 449 had valid accelerometry data. Complete datasets ranged from 408 (hip DXA), 406 (tibial pQCT) and 297 (bone turnover markers) (see Fig. 1). Based on individuals with complete hip DXA results, women were a mean age of 76.7 years, and wore accelerometers for a mean of 5.4 days (Table 1). A median of 8809, 345 and 42 low, medium and higher impacts per week were observed, respectively. COSHIBA participants who were invited to participate but did not attend a clinic session were a mean of 80.7 years of age.

**Table 1:** Descriptive statistics

	Number	Median	p25	p75
Age (years)	408	76.2	74.6	78.1
Height (cm)	408	159.1	154.6	162.8
Weight (kg)	408	67.0	59.8	76.1
Accelerometer wear time (valid days)	408	6.0	5.0	6.0
Low band peaks (≥0.5 to <1.0 g)	408	8809	4047	16,882
Medium band peaks (≥1.0 to <1.5 g)	408	345	99	764
High band peaks ≥1.5 g	408	42	17	106
Tibial pQCT measures				
PC (mm)	406	69.8	67.4	73.2
CT (mm)	406	4.8	4.2	5.4
BMD_c_ (mg/cm^3^)	406	1121	1090	1146
BMD_t_ (mg/cm^3^) (4%)	401	306	292	326
CSMI (mm^4^)	406	10,567	9131	12,251
SSI (mm^3^)	406	885	782	989
Radial pQCT measures				
PC (mm)	401	37.9	36.2	40.0
CT (mm)	401	2.4	2.1	2.8
BMD_c_ (mg/cm^3^)	401	1104	1070	1127
BMD_t_ (mg/cm^3^) (4 %)	423	332	322	343
CSMI (mm^4^)	401	892	705	1107
SSI (mm^3^)	401	134.8	113.2	162.4
DXA measures				
FM (kg)	408	27.2	22.2	32.9
LM (kg)	408	37.6	34.6	41.0
TH BMD (g/cm^2^)	408	0.86	0.78	0.97
FN (g/cm2)	408	0.82	0.74	0.92
MNW (mm)	408	30.6	29.1	31.80
CSMI (cm^4^)	408	9.1	7.6	10.5
LS BMD (g/cm^2^)	401	1.0	0.89	1.2
Bone turnover markers				
β-CTX (μg/L)	293	0.39	0.27	0.53
P1NP (μg/L)	293	42.6	32.6	52.5

Table shows participant characteristics including DXA measures [*FM* fat mass, *LM* lean mass, *TH* total hip, *FN* femoral neck and *LS* lumbar spine BMD, *MNW* minimum femoral neck width and *CSMI* cross sectional moment of inertia ], pQCT [*PC* periosteal circumference, *CT* cortical thickness (CT), *BMD*_*c*_ cortical BMD, *BMD*_*t*_ trabecular density, *CSMI* cross-sectional moment of inertia and *SSI* strength strain index], and bone turnover markers [*β-CTX* C-terminal telopeptide and *P1NP* propeptide of type I collagen]

### Impacts versus pQCT parameters

Linear regression analyses were performed to examine, in turn, the associations between the number of low, medium and high vertical impacts recorded for each individual and bone measures. Higher impacts were positively associated with tibial PC, CSMI and SSI in all models, with strongest associations observed in our fully adjusted model (model 3) [tibial PC 0.015 (0.003, 0.027); CSMI 0.042 (0.012, 0.072); SSI 0.034 (0.009, 0.058)] (Table 2). Low and medium impacts were unrelated to these parameters.

**Table 2:** Associations between vertical impacts and tibia pQCT measures

Tibia pQCT variable	Model	Low impacts				Medium impacts				Higher impacts			
		Beta	Lower CI	Upper CI	*p*	Beta	Lower CI	Upper CI	*p*	Beta	Lower CI	Upper CI	*p*
PC (mm)	Model 1	0.000	−0.011	0.011	0.991	0.003	−0.005	0.011	0.485	0.010	0.001	0.018	0.022
	Model 2	0.002	−0.009	0.013	0.686	0.004	−0.004	0.011	0.347	0.009	0.001	0.016	0.022
	Model 3	−0.002	−0.021	0.018	0.866	−0.007	−0.024	0.010	0.436	0.015	0.003	0.027	0.017
CT (mm)	Model 1	−0.014	−0.039	0.011	0.264	−0.014	−0.032	0.004	0.128	0.001	−0.018	0.020	0.936
	Model 2	0.011	−0.016	0.038	0.412	−0.004	−0.023	0.014	0.629	0.002	−0.016	0.021	0.794
	Model 3	0.058	0.010	0.105	0.018	−0.053	−0.095	−0.011	0.013	0.022	−0.008	0.051	0.145
BMD_c_ (mg/m^3^)	Model 1	−0.014	−0.029	0.000	0.050	−0.010	−0.021	0.000	0.055	−0.011	−0.022	0.000	0.057
	Model 2	−0.008	−0.024	0.008	0.330	−0.007	−0.018	0.004	0.205	−0.009	−0.021	0.002	0.095
	Model 3	−0.001	−0.030	0.028	0.926	0.001	−0.025	0.027	0.925	−0.010	−0.028	0.008	0.281
BMD_t_ (mg/cm^3^)	Model 1	−0.081	−0.143	−0.019	0.010	−0.066	−0.110	−0.022	0.004	−0.052	−0.099	−0.006	0.028
	Model 2	−0.032	−0.101	0.038	0.370	−0.043	−0.090	0.003	0.067	−0.041	−0.088	0.006	0.086
	Model 3	0.062	−0.061	0.186	0.322	−0.070	−0.179	0.039	0.207	−0.010	−0.086	0.066	0.799
CSMI (mm^4^)	Model 1	0.000	−0.029	0.028	0.984	0.006	−0.015	0.026	0.595	0.026	0.005	0.048	0.017
	Model 2	0.013	−0.014	0.040	0.345	0.010	−0.008	0.029	0.282	0.024	0.005	0.042	0.012
	Model 3	0.016	−0.033	0.064	0.524	−0.031	−0.073	0.012	0.159	0.042	0.012	0.072	0.006
SSI (mm^3^)	Model 1	−0.008	−0.032	0.016	0.495	−0.001	−0.019	0.016	0.884	0.017	−0.001	0.036	0.058
	Model 2	0.007	−0.016	0.029	0.554	0.004	−0.011	0.019	0.578	0.016	0.001	0.031	0.038
	Model 3	0.015	−0.024	0.055	0.449	−0.030	−0.065	0.005	0.090	0.034	0.009	0.058	0.007

Table shows associations between number of low (0.5–1 g), medium (1–1.5 g) and higher (>1.5 g) impacts normalised to 7 days, and tibial pQCT measures comprising periosteal circumference (PC), cortical thickness (CT), cortical BMD (BMDc), trabecular density (BMDt) cross sectional moment of inertia (CSMI) and strength strain index (SSI) in 406 participants (*n* = 401 for trabecular density). Beta shows SD change in outcome per doubling in number of impacts. *Model 1* adjusted for age and error grade, *model 2* adjusted for age, error grade, height, fat and lean mass, *model 3* as for model 2 plus adjustment for other bands

High, medium and low impacts were inversely related to BMD_c_ and BMD_t_ in analyses adjusted for age only (model 1); however, these associations were largely attenuated by further adjustment for height and body composition (models 2 and 3). No associations were observed between impacts and CT in models 1 and 2. However, in model 3 (further adjustment for other bands), low and medium impacts showed inverse associations with CT. With the exception of a positive association between low-impact PA and radial BMD_c_ in model 3, little relationship was observed between impacts and radial pQCT parameters (supplementary Table 1).

### Impacts versus DXA parameters

Higher impacts were unrelated to CSMI in analyses models 1 or 2 (Table 3). However, higher impacts were positively associated with CSMI in model 3 [0.067 (0.001, 0.133)] (beta coefficient and 95 % confidence interval). Higher impacts were unrelated to other hip geometric parameters, to LS BMD, and to TH and FN BMD, in all models. Medium impacts were inversely related to CSMI in all models, to MNW in model 2 only, and to LS BMD only in model 1. Low impacts showed a similar pattern in that they were inversely related to CSMI (models 1 and 2), MNW (model 2) and LS BMD (model 1). In addition, low impacts were inversely related to TH BMD [−0.071 (−0.134, −0.008)] and a lesser extent FN BMD [−0.059 (−0.122, 0.003)].

**Table 3:** Associations between vertical impacts and DXA measures

		Low impacts				Medium impacts				Higher impacts			
		Beta	Lower CI	Upper CI	*p*	Beta	Lower CI	Upper CI	*p*	Beta	Lower CI	Upper CI	*p*
TH BMD (g/cm^2^)	Model 1	−0.071	−0.134	−0.008	0.027	−0.034	−0.080	0.012	0.147	0.001	−0.048	0.050	0.963
	Model 2	0.043	−0.021	0.108	0.188	0.015	−0.028	0.059	0.492	0.020	−0.025	0.065	0.383
	Model 3	0.087	−0.030	0.203	0.145	−0.055	−0.158	0.047	0.287	0.030	−0.042	0.101	0.417
FN (g/cm2)	Model 1	−0.059	−0.122	0.003	0.063	−0.036	−0.082	0.009	0.119	−0.006	−0.055	0.042	0.798
	Model 2	0.012	−0.055	0.078	0.731	−0.007	−0.052	0.039	0.773	0.005	−0.042	0.051	0.843
	Model 3	0.072	−0.048	0.192	0.237	−0.072	−0.177	0.033	0.180	0.033	−0.041	0.107	0.380
MNW (mm)	Model 1	−0.050	−0.115	0.015	0.129	−0.035	−0.082	0.012	0.143	−0.010	−0.060	0.040	0.691
	Model 2	−0.076	−0.138	−0.014	0.017	−0.050	−0.092	−0.008	0.020	−0.021	−0.064	0.023	0.349
	Model 3	−0.033	−0.145	0.080	0.567	−0.064	−0.162	0.035	0.203	0.043	−0.027	0.112	0.227
CSMI (cm^4^)	Model 1	−0.090	-0.152	−0.027	0.005	−0.060	−0.105	−0.014	0.010	−0.015	−0.064	0.033	0.534
	Model 2	−0.069	−0.128	−0.009	0.024	−0.053	−0.093	−0.013	0.010	−0.015	−0.056	0.027	0.489
	Model 3	0.008	−0.099	0.115	0.882	−0.108	−0.202	−0.014	0.025	0.067	0.001	0.133	0.045
LS BMD (g/cm^2^)	Model 1	−0.161	−0.227	−0.095	0.000	−0.091	−0.139	−0.043	0.000	−0.032	−0.084	0.019	0.220
	Model 2	−0.059	−0.127	0.010	0.096	−0.040	−0.087	0.006	0.091	−0.007	−0.055	0.041	0.783
	Model 3	−0.012	−0.137	0.113	0.851	−0.082	−0.191	0.028	0.143	0.064	−0.014	0.141	0.107

Table shows associations between number of low (0.5–1 g), medium (1–1.5 g) and higher (>1.5 g) impacts normalised to 7 days, and DXA measures comprising total hip (TH), femoral neck (FN) and lumbar spine (LS) BMD, minimum femoral neck width (MNW) and cross sectional moment of inertia (CSMI), in 408 participants (*n* = 401 for LS BMD). Beta shows SD change in outcome per doubling in number of impacts. *Model 1* adjusted for age, *model 2* adjusted for age, height, fat and lean mass, *model 3* as for model 2 plus adjustment for other bands

### Impacts versus β-CTX and P1NP

Higher impacts were positively related to β-CTX in model 1 [0.101 (0.040, 0.162)], which was slightly attenuated in model 2 [0.088 (0.026, 0.150), and further attenuated in model 3 [0.085 (−0.017, 0.187)] (Table 4). Medium and low impacts showed a similar positive association with β-CTX compared to that seen high impacts, with progressive attenuation with further adjustment. Higher impacts were positively related to P1NP in all models with strongest associations observed in model 3 [0.127 (0.031, 0.224)]. Weaker associations were observed between low and medium impacts and P1NP in models 1 and 2, whereas these associations were fully attenuated in model 3.

**Table 4:** Associations between vertical impacts and bone turnover markers

		Low impacts				Medium impacts				Higher impacts			
		Beta	Lower CI	Upper CI	*p*	Beta	Lower CI	Upper CI	*p*	Beta	Lower CI	Upper CI	*p*
β-CTX (μg/L)	Model 1	0.101	0.019	0.183	0.016	0.089	0.031	0.148	0.003	0.101	0.040	0.162	0.001
	Model 2	0.054	−0.037	0.144	0.245	0.066	0.004	0.127	0.036	0.088	0.026	0.150	0.006
	Model 3	−0.048	−0.206	0.111	0.556	0.027	−0.117	0.171	0.713	0.085	−0.017	0.187	0.103
P1NP (μg/L)	Model 1	0.049	−0.028	0.126	0.214	0.056	0.000	0.111	0.048	0.095	0.038	0.152	0.001
	Model 2	0.036	−0.050	0.122	0.412	0.048	−0.011	0.106	0.110	0.088	0.029	0.147	0.003
	Model 3	−0.021	-0.172	0.129	0.780	−0.039	−0.174	0.097	0.577	0.127	0.031	0.224	0.010

Table shows associations between number of low (0.5–1 g), medium (1–1.5 g) and higher (>1.5 g) impacts normalised to 7 days, and C-terminal telopeptide (β-CTX) and propeptide of type I collagen (P1NP) in 297 participants. Beta shows SD change in outcome per doubling in number of impacts. *Model 1* adjusted for age and sample timing (am/pm), *model 2* adjusted for age, height, fat and lean mass, *model 3* as for model 2 plus adjustment for other bands

### Sensitivity analyses and further confounder adjustment

Similar results were obtained when analyses were restricted to participants with accelerometry data collected for a minimum of three valid days (results not shown). Further models were explored to analyse the role of different confounders, in 373 participants in whom additional information was available from questionnaires on socioeconomic status as reflected by SOC 90, co-morbidities and concurrent use of bone-active medication (supplementary Table 2). Higher impacts showed similar relationships with tibial PC and CSMI to those seen in the wider cohort, with little change in beta coefficients following adjustment for any of the confounders examined (supplementary Table 3). We also examined the role of past weight-bearing PA, but no relationship was evident between levels of impacts >1.5 g and extent of self-reported participation in weight-bearing PA <18 years, or 18–29 years, and results were unaffected by adjustment for past activity (results not shown). Finally, we examined whether associations between impacts and bone turnover markers might explain those with tibial pQCT parameters. Higher impacts showed equivalent associations with tibial PC and CSMI in the subset of participants in whom bone turnover marker data were also available, as compared with the larger sample shown in Table 3; relationships were unaffected by additional adjustment for β-CTX or P1NP (supplementary Table 4).

## Discussion

We examined the relationship between PA and bone health measures in a population-based cohort of older women, based on classification of vertical movements from accelerometers according to level of impact. Based on our fully adjusted model, we found that higher, but not medium or low, vertical impacts were positively related to estimated bone strength as reflected by hip and tibial CSMI, and tibial SSI. The associations we observed with lower limb strength appeared to largely reflect changes in overall bone size since higher impacts were also positively related to tibial PC, whereas little relationship was evident with BMD. Taken together, our findings are consistent with the possibility that, despite their rarity and the relatively low *g* threshold used to define them, habitual exposure to higher vertical impacts in older women is associated with greater lower limb size and strength.

In contrast to higher impacts, low/medium impacts were inversely related to several bone measures. In view of the opposing relationship between low and high impacts and certain bone parameters, and the correlation that exists between impacts in different bands, there was a strong rationale for including adjustment for other bands in our final model. As expected, associations between higher impacts and tibial PC and CSMI were strengthened after adjustment for other impacts. Moreover, a positive association between higher impacts and hip CSMI only became evident after adjustment for low and medium impacts, consistent with the inverse association between low and medium impacts and hip CSMI despite body composition adjustment.

Our observation that higher, as opposed to medium or low, vertical impacts are related to lower limb bone strength is consistent with results of previous interventional studies, as judged by meta-analyses indicating positive effects of high, but not low, impact PA interventions on BMD [3, 18, 19]. In observational studies, equivalent positive relationships between day-to-day participation in high-impact PA and lower limb bone strength has previously been reported in young and middle-aged adults based on PA questionnaires in which different activities are graded according to impact level [9, 10], but limited data is available in older populations. Johansson et al. employed actigraph accelerometers to examine relationships between PA and skeletal health in a mixed population aged 70 years, using counts per minute thresholds calibrated according to metabolic rate to define PA intensity; whereas a positive association was observed between moderate PA and tibial PC, no association was observed for vigorous PA [20]. In contrast, previous studies using Newtest accelerometers in which peak accelerations are classified according to *g* level have yielded similar findings to those reported here in that only high vertical impacts were found to be related to lower limb bone outcomes in adolescents and premenopausal women [13, 14, 21].

Individuals whose higher impact counts were in the top quartile (i.e. greater than 106 counts per weeks) experienced approximately fourfold more high impacts compared with those in the bottom quartile (i.e. less than 17 counts per week), which translates into a 0.13 SD greater hip CSMI. Since greater hip CSMI is an established protective factor for hip fracture [22], this association between higher impacts and CSMI is expected to lead to a reduced risk of hip fracture. However, no association was observed with hip BMD, which is more strongly related to hip fracture risk compared with hip CSMI [22]. Therefore, although our findings support our prior hypothesis that positive relationships between day-to-day PA and bone strength in older women are explained by exposure to higher impacts, any benefit in terms of fracture prevention is likely to be limited.

A possible explanation for this limited association is that the quartile of our study population with the greatest number of high impacts only experienced approximately 15 vertical impacts >1.5 g daily. In terms of the activities responsible for high impacts, 1.5 g exceeds that associated with walking (typically 0.5–1.0 g), but is achieved in the majority of aerobics class exercises undertaken by older individuals, particularly those with a jumping component [23]. Aerobics exercise participation was one of the commonest forms of high-impact PA in COSHIBA as assessed by questionnaire, and presumably contributed to the positive relationship we observed between impacts >1.5 g and lower limb bone strength [24]. Alternatively, the weak associations we observed could be a reflection of the relatively low levels of impact involved, and it may be that stronger impacts, closer to the 4-g osteogenic threshold identified in studies of younger individuals, are required to produce a significant benefit in terms of bone strength and fracture risk. Though impacts of this level are rarely achieved in unselected individuals, in our previous exercise study, we found that impacts within this range are readily achievable with only minor modifications to the class protocol [23].

Our fully adjusted model suggested that higher, but not medium or low, impacts exert a net positive effect on bone formation, as reflected by a positive association with P1NP but not β-CTX. Although we speculated that this might represent a mechanism by which higher impacts improve bone strength, these associations were found to be unaffected by P1NP adjustment. Few previous observational studies have examined relationships between day-to-day levels of PA and bone markers. Our results are potentially consistent with a previous study of 530 premenopausal women, in which weight-bearing PA, as assessed by questionnaire, was found to be positively related to P1NP levels, whereas no association was observed with β-CTX; however, these relationships were not broken down according to PA intensity or type [25].

Some of the inverse associations we observed between low/medium impacts and bone measures were attenuated after adjustment for body composition, presumably reflecting the fact that PA is known to be inversely related to fat mass, of which the latter is positively related to BMD involving a causal pathway between fat and bone [26]. Interventional studies provide further evidence that weight loss including low-intensity exercise has a net adverse effect on BMD [27]. However, several of these low/medium impact inverse associations, e.g. with MNW and tibial CSMI, persisted following adjustment for body composition. Whereas older individuals participating in more low and moderate impact PA may have had a lower fat mass over their life course, leading to long-term effects on their skeleton, this may be poorly reflected by body composition measurements taken at a mean of 77 years of age. Alternatively, it may be that low or medium impacts exert hitherto unrecognised effects on bone metabolism. For example, in our minimally adjusted analyses, low impacts were positively associated with the bone resorption marker *β*-CTX, whereas no association was observed with the formation marker P1NP, suggesting a net balance of resorption over formation leading to bone loss.

### Limitations

There are several inherent limitations in use of accelerometry to measure day-to-day PA. For example, an individual’s daily activity may be altered as a result of being recorded. In addition, PA levels are likely to be affected by seasonal influences. However, seasonal variation in weather patterns in the southwest of the UK is limited, and any effect on PA levels would have reduced the strength of associations by increasing variability, rather than leading to spurious associations through introducing bias. In terms of other limitations, our cross-sectional study design limits the ability to infer causality, and we are unable to distinguish whether the association we observed between higher impacts and bone strength reflects an osteogenic effect of high-impact PA on bone, or arises from confounding. That said, our results were unaffected by adjustment for a range of confounders ascertained by contemporaneous questionnaire. Another important limitation is that given the relatively small effect sizes observed, and the multiple outcomes and models examined, we are unable to exclude the possibility of a type I error. A further limitation is the generalisability of our results to the wider population. Although our study was based on a population-based cohort, 44 % of those invited attended the research clinic, which is likely to have resulted in selection of a relatively healthy sub-group, consistent with which those attending were approximately 4 years younger than those who did not attend.

## Conclusions

We investigated relationships between vertical acceleration peaks as measured by 7-day accelerometer recordings and lower limb skeletal strength, in a population-based cohort of older women. Despite their rarity and the relatively low 1.5 g threshold, day-to-day levels of higher vertical impacts were positively associated with lower limb bone size and strength, whereas equivalent associations were not seen for medium or low impacts. These findings are consistent with the possibility that despite the low levels of impacts achieved, day-to-day PA in older women is beneficial for their skeletal health.

## Electronic supplementary material

ESM 1 (DOCX 26 kb)
