# Supplementary material for: Habitual levels of higher, but not medium or low, impact physical activity are positively related to lower limb bone strength in older women: findings from a population-based study using accelerometers to classify impact magnitude
Source: Osteoporos Int. 2016 Dec 13;28(10):2813–22. doi: 10.1007/s00198-016-3863-5 (PMC5624975; doi:10.1007/s00198-016-3863-5)
Supplement: Supplementary file 1 — (DOCX 26 kb) [file 198_2016_3863_MOESM1_ESM.docx]

**Supplementary material**

**Osteoporosis International**

**Habitual levels of higher, but not medium or low, impact physical activity are positively related to lower limb bone strength in older women: findings from a population based study using accelerometers to classify impact magnitude**

K Hannam^1^, KC Deere^1^, A Hartley^1^, UA Al-Sari^1^, EM Clark^1^, WD Fraser^2^, JH Tobias^1^

^1^Musculoskeletal Research Unit, University of Bristol School of Clinical Sciences

^2^University of East Anglia

Corresponding author:-

Professor Jon Tobias,

Musculoskeletal Research Unit, University of Bristol School of Clinical Sciences, Southmead Hospital, Bristol BS10 5NB, UK

Jon.Tobias@bristol.ac.uk

Kimberly Hannam, Kevin Deere, April Hartley, Usama Al-Sari, Emma Clark, William Fraser and Jon Tobias declare that they have no conflict of interest.

**Supplementary Table 1: Associations between physical activity impacts and radial pQCT**

|  |  | **Low Impacts** | | | | **Medium Impacts** | | | | **Higher Impacts** | | | |
| --- | --- | --- | --- | --- | --- | --- | --- | --- | --- | --- | --- | --- | --- |
|  |  | **Beta** | **lower CI** | **upper CI** | **p** | **Beta** | **lower CI** | **upper CI** | **p** | **Beta** | **lower CI** | **upper CI** | **p** |
| **PC (mm)** | *Model 1* | 0.003 | -0.011 | 0.017 | 0.700 | 0.006 | -0.004 | 0.016 | 0.261 | 0.010 | -0.001 | 0.020 | 0.076 |
|  | *Model 2* | 0.007 | -0.008 | 0.021 | 0.364 | 0.006 | -0.004 | 0.015 | 0.228 | 0.008 | -0.002 | 0.017 | 0.126 |
|  | *Model 3* | 0.000 | -0.025 | 0.025 | 0.976 | 0.000 | -0.022 | 0.022 | 0.996 | 0.007 | -0.008 | 0.023 | 0.353 |
| **CT (mm)** | *Model 1* | -0.020 | -0.053 | 0.012 | 0.219 | -0.018 | -0.042 | 0.005 | 0.124 | -0.010 | -0.035 | 0.014 | 0.413 |
|  | *Model 2* | 0.009 | -0.027 | 0.044 | 0.633 | -0.008 | -0.031 | 0.016 | 0.526 | -0.007 | -0.031 | 0.017 | 0.578 |
|  | *Model 3* | 0.056 | -0.007 | 0.118 | 0.080 | -0.042 | -0.097 | 0.013 | 0.134 | 0.004 | -0.035 | 0.043 | 0.828 |
| **BMD_c_ (mg/m^3^)** | *Model 1* | 0.010 | -0.011 | 0.032 | 0.344 | -0.003 | -0.018 | 0.013 | 0.755 | -0.003 | -0.019 | 0.014 | 0.765 |
|  | *Model 2* | 0.012 | -0.013 | 0.036 | 0.351 | -0.004 | -0.020 | 0.013 | 0.668 | -0.003 | -0.020 | 0.014 | 0.693 |
|  | *Model 3* | 0.050 | 0.007 | 0.093 | 0.023 | -0.035 | -0.073 | 0.003 | 0.073 | 0.004 | -0.023 | 0.031 | 0.751 |
| **BMD_t_ (mg/cm^3^)** | *Model 1* | 0.000 | -0.055 | 0.056 | 0.988 | 0.011 | -0.029 | 0.051 | 0.580 | 0.013 | -0.029 | 0.055 | 0.551 |
|  | *Model 2* | -0.044 | -0.106 | 0.018 | 0.164 | -0.007 | -0.049 | 0.035 | 0.730 | 0.003 | -0.040 | 0.046 | 0.894 |
|  | *Model 3* | -0.103 | -0.213 | 0.006 | 0.065 | 0.043 | -0.054 | 0.140 | 0.383 | 0.009 | -0.059 | 0.078 | 0.786 |
| **CSMI (mm^4^)** | *Model 1* | -0.001 | -0.037 | 0.035 | 0.961 | 0.007 | -0.018 | 0.033 | 0.575 | 0.020 | -0.008 | 0.047 | 0.157 |
|  | *Model 2* | 0.017 | -0.019 | 0.053 | 0.357 | 0.011 | -0.013 | 0.035 | 0.373 | 0.016 | -0.009 | 0.041 | 0.208 |
|  | *Model 3* | 0.016 | -0.047 | 0.080 | 0.614 | -0.013 | -0.069 | 0.043 | 0.648 | 0.020 | -0.020 | 0.060 | 0.330 |
| **SSI (mm^3^)** | *Model 1* | -0.002 | -0.047 | 0.043 | 0.930 | 0.003 | -0.030 | 0.036 | 0.852 | 0.018 | -0.016 | 0.053 | 0.301 |
|  | *Model 2* | 0.022 | -0.024 | 0.068 | 0.351 | 0.008 | -0.023 | 0.040 | 0.607 | 0.014 | -0.018 | 0.046 | 0.379 |
|  | *Model 3* | 0.044 | -0.038 | 0.126 | 0.295 | -0.035 | -0.107 | 0.038 | 0.345 | 0.024 | -0.027 | 0.076 | 0.348 |

Table shows associations between number of low (0.5-1g), medium (1-1.5g) and higher (>1.5g) impacts normalised to 7 days, and radial pQCT measures comprising periosteal circumference (PC), cortical thickness (CT), cortical BMD (BMD_c_), trabecular density (BMD_t_) cross sectional moment of inertia (CSMI) and strength strain index (SSI) and in 401 participants (n=423 for trabecular density). Beta shows SD change in outcome per doubling in number of impacts. Model 1 = adjusted for age and error grade, model 2 = adjusted for age, error grade, height, fat and lean mass, model 3 = as for model 2 plus adjustment for other bands

**Supplementary table 2: Descriptive statistics for additional confounders**

|  | **N** | **%** |
| --- | --- | --- |
| **Social class (SOC90 code)** |  |  |
| 1. managers & administrators | 80 | 21.1 |
| 2. professional | 111 | 29.3 |
| 3. associate professional & technical | 42 | 11.1 |
| 4. clerical & secretarial | 77 | 20.3 |
| 5. craft and related | 30 | 7.9 |
| 6. personal & protective services | 21 | 5.5 |
| 7. sales | 8 | 2.1 |
| 8. plant & machine operatives | 8 | 2.1 |
| 9. other occupations | 2 | 0.53 |
| **Sum of comorbidities** |  |  |
| 0 | 141 | 37.2 |
| 1 | 171 | 45.1 |
| 2 | 57 | 15.0 |
| 3 | 9 | 2.4 |
| 4 | 1 | 0.26 |
| **Bone active medication** |  |  |
| No | 345 | 91.0 |
| Yes | 34 | 9.0 |

Table shows descriptive statistics for the additional confounders taken from questionnaires, restricted to the 373 participants included in further analyses of the pQCT variables as shown in Table 4. Co-morbidities comprise respiratory disease (14.8%), cardiovascular disease (12.4%), arterial disease (2.4%), stroke (5.8%), Parkinson’s (0.3%) and arthritis (48.0%).

**Supplementary Table 3: Associations between physical activity impacts and tibia pQCT measures adjusted for additional confounders**

|  |  | **Low Impacts** | | | | **Medium Impacts** | | | | **Higher Impacts** | | | |
| --- | --- | --- | --- | --- | --- | --- | --- | --- | --- | --- | --- | --- | --- |
| **Tibia pQCT variable** | **Model** | **Beta** | **lower CI** | **upper CI** | **p** | **Beta** | **lower CI** | **upper CI** | **p** | **Beta** | **lower CI** | **upper CI** | **p** |
| **PC (mm)** | *Model 1* | 0.002 | -0.010 | 0.014 | 0.714 | 0.004 | -0.004 | 0.013 | 0.313 | 0.011 | 0.002 | 0.020 | 0.019 |
|  | *Model 2* | 0.005 | -0.006 | 0.017 | 0.379 | 0.006 | -0.002 | 0.013 | 0.169 | 0.009 | 0.001 | 0.017 | 0.021 |
|  | *Model 3* | 0.000 | -0.020 | 0.021 | 0.965 | -0.004 | -0.023 | 0.014 | 0.632 | 0.012 | 0.000 | 0.025 | 0.056 |
|  | *Model 4* | 0.000 | -0.021 | 0.020 | 0.973 | -0.004 | -0.023 | 0.014 | 0.633 | 0.012 | 0.000 | 0.025 | 0.055 |
|  | *Model 5* | 0.001 | -0.020 | 0.021 | 0.958 | -0.004 | -0.023 | 0.014 | 0.632 | 0.012 | 0.000 | 0.025 | 0.055 |
|  | *Model 6* | 0.000 | -0.020 | 0.020 | 0.972 | -0.004 | -0.023 | 0.014 | 0.641 | 0.014 | 0.002 | 0.027 | 0.028 |
| **CSMI (mm^4^)** | *Model 1* | 0.007 | -0.024 | 0.037 | 0.655 | 0.011 | -0.011 | 0.033 | 0.343 | 0.030 | 0.007 | 0.052 | 0.010 |
|  | *Model 2* | 0.023 | -0.006 | 0.052 | 0.117 | 0.017 | -0.003 | 0.036 | 0.098 | 0.026 | 0.007 | 0.046 | 0.008 |
|  | *Model 3* | 0.023 | -0.027 | 0.073 | 0.364 | -0.026 | -0.071 | 0.020 | 0.269 | 0.037 | 0.006 | 0.069 | 0.020 |
|  | *Model 4* | 0.021 | -0.030 | 0.072 | 0.412 | -0.026 | -0.071 | 0.020 | 0.269 | 0.038 | 0.006 | 0.069 | 0.020 |
|  | *Model 5* | 0.023 | -0.027 | 0.073 | 0.368 | -0.026 | -0.071 | 0.020 | 0.270 | 0.037 | 0.006 | 0.069 | 0.021 |
|  | *Model 6* | 0.022 | -0.028 | 0.073 | 0.383 | -0.026 | -0.071 | 0.020 | 0.272 | 0.040 | 0.008 | 0.072 | 0.015 |

Table shows associations between number of low (0.5-1g), medium (1-1.5g) and higher (>1.5g) impacts normalised to 7 days, and tibial periosteal circumference (PC) and cross sectional moment of inertia (CSMI) in 373 participants with available data on confounders as assessed by questionnaire. Beta shows SD change in outcome per doubling in number of impacts. Model 1 = adjusted for age and error grade, model 2 = adjusted for age, error grade, height, fat and lean mass, model 3 = model 2 plus adjustment for other bands, model 4= model 3 plus comorbidities, model 5= model 3 plus SES, model 6= model 3 plus bone active medication.

**Supplementary Table 4:** **Associations between physical activity impacts and tibia pQCT measures with adjustment for bone turnover markers**

|  |  | **Low Impacts** | | | | **Medium Impacts** | | | | **Higher Impacts** | | | |
| --- | --- | --- | --- | --- | --- | --- | --- | --- | --- | --- | --- | --- | --- |
| **Tibia pQCT variable** | **Model** | **Beta** | **lower CI** | **upper CI** | **p** | **Beta** | **lower CI** | **upper CI** | **p** | **Beta** | **lower CI** | **upper CI** | **p** |
| **PC (mm)** | *Model 1* | 0.000 | -0.014 | 0.013 | 0.951 | 0.005 | -0.004 | 0.015 | 0.274 | 0.011 | 0.001 | 0.021 | 0.033 |
|  | *Model 2* | 0.000 | -0.013 | 0.013 | 0.990 | 0.005 | -0.004 | 0.014 | 0.313 | 0.009 | 0.000 | 0.018 | 0.053 |
|  | *Model 3* | -0.013 | -0.036 | 0.010 | 0.258 | 0.003 | -0.018 | 0.024 | 0.776 | 0.012 | -0.003 | 0.026 | 0.113 |
|  | *Model 4* | -0.013 | -0.036 | 0.010 | 0.274 | 0.003 | -0.018 | 0.023 | 0.796 | 0.011 | -0.003 | 0.026 | 0.130 |
|  | *Model 5* | -0.013 | -0.036 | 0.010 | 0.272 | 0.003 | -0.017 | 0.024 | 0.749 | 0.010 | -0.004 | 0.025 | 0.164 |
| **CSMI (mm^4^)** | *Model 1* | -0.005 | -0.039 | 0.029 | 0.788 | 0.009 | -0.016 | 0.033 | 0.479 | 0.027 | 0.002 | 0.052 | 0.036 |
|  | *Model 2* | 0.005 | -0.027 | 0.038 | 0.744 | 0.010 | -0.012 | 0.033 | 0.365 | 0.023 | 0.001 | 0.045 | 0.043 |
|  | *Model 3* | -0.010 | -0.067 | 0.048 | 0.741 | -0.013 | -0.064 | 0.039 | 0.627 | 0.037 | 0.001 | 0.073 | 0.046 |
|  | *Model 4* | -0.010 | -0.067 | 0.048 | 0.736 | -0.013 | -0.064 | 0.039 | 0.632 | 0.037 | 0.001 | 0.073 | 0.046 |
|  | *Model 5* | -0.009 | -0.067 | 0.048 | 0.748 | -0.012 | -0.064 | 0.039 | 0.635 | 0.036 | -0.001 | 0.072 | 0.055 |

Table shows associations between number of low (0.5-1g), medium (1-1.5g) and higher (>1.5g) impacts normalised to 7 days, and tibial periosteal circumference (PC) and cross sectional moment of inertia (CSMI) in 295 participants. Beta shows SD change in outcome per doubling in number of impacts. Model 1 = adjusted for age and error grade, model 2 = adjusted for age, error grade, height, fat and lean mass, model 3 = as for model 2 plus adjustment for other bands, model 4 = as for model 3 plus adjustment for β-CTX, model 5 = as for model 3 plus adjustment for P1NP
